# Designing Probiotic Therapies With Broad-Spectrum Activity Against a Wildlife Pathogen

**DOI:** 10.3389/fmicb.2019.03134

**Published:** 2020-01-22

**Authors:** Xavier A. Harrison, Thomas Sewell, Matthew Fisher, Rachael E. Antwis

**Affiliations:** ^1^Institute of Zoology, Zoological Society of London, London, United Kingdom; ^2^Centre for Ecology and Conservation, University of Exeter, Penryn, United Kingdom; ^3^MRC Centre for Global Infectious Disease Analysis, School of Public Health, Imperial College London, London, United Kingdom; ^4^School of Science, Engineering and Environment, University of Salford, Salford, United Kingdom

**Keywords:** chytrid fungus, *Batrachochytrium dendrobatidis*, wildlife disease, amphibian decline, amphibian disease

## Abstract

Host-associated microbes form an important component of immunity that protect against infection by pathogens. Treating wild individuals with these protective microbes, known as probiotics, can reduce rates of infection and disease in both wild and captive settings. However, the utility of probiotics for tackling wildlife disease requires that they offer consistent protection across the broad genomic variation of the pathogen that hosts can encounter in natural settings. Here we develop multi-isolate probiotic consortia with the aim of effecting broad-spectrum inhibition of growth of the lethal amphibian pathogen *Batrachochytrium dendrobatidis* (*Bd*) when tested against nine *Bd* isolates from two distinct lineages. Though we achieved strong growth inhibition between 70 and 100% for seven *Bd* isolates, two isolates appeared consistently resistant to inhibition, irrespective of probiotic strategy employed. We found no evidence that genomic relatedness of the chytrid predicted similarity of inhibition scores, nor that increasing the genetic diversity of the bacterial consortia could offer stronger inhibition of pathogen growth, even for the two resistant isolates. Our findings have important consequences for the application of probiotics to mitigate wildlife diseases in the face of extensive pathogen genomic variation.

## Introduction

Emerging infectious diseases pose a significant threat to human and wildlife health, and are responsible for driving declines in biodiversity around the globe (Jones et al., 2008; Tompkins et al., 2015; Scheele et al., 2019). The last few decades alone have witnessed the emergence and spread of multiple viral and fungal pathogens capable of infecting a broad range of host species, and developing strategies to mitigate the loss of wildlife species caused by disease is now a key priority. The use of probiotics, where hosts are exposed to microbes with known host-protective properties, represent a promising tool for tackling infectious disease (McKenzie et al., 2018). To date, probiotics have been applied in the fields of human medicine (Kerry et al., 2018), wildlife medicine (Kueneman et al., 2016), aquaculture (Tan et al., 2016) and both arable (Marcano et al., 2016) and livestock (Mingmongkolchai and Panbangred, 2018) agriculture, with varying success. Developing effective probiotics is complicated by the fact that distinct lineages of a pathogen exhibit markedly different susceptibilities to the same bacterial assemblages (Antwis et al., 2015; Antwis and Weldon, 2017; Muletz-Wolz et al., 2017; Antwis and Harrison, 2018). A bacterial probiotic that is inhibitory for one variant of a pathogen can promote growth of a different variant (e.g., Antwis and Harrison, 2018). Therefore, probiotics comprising single bacterial species appear unlikely to be provide a “silver bullet” capable of inhibition across the broad spectrum of pathogen variants observed in natural systems. This issue is exacerbated by the propensity of pathogen genotypes to spread rapidly around the globe, often as a result of human activity (O’Hanlon et al., 2018), exposing susceptible hosts to multiple and novel threats (Greenspan et al., 2018; Longo et al., 2019). Therefore, designing novel probiotic strategies that are effective against the full panorama of pathogen genotypes is necessary in order to effect meaningful change in the rates of infection caused by emerging infectious diseases.

Recent evidence suggests that the shortcomings of single species probiotics can be overcome by administering multiple bacterial isolates in a mixed community, referred to as a consortium (e.g., Piovia-Scott et al., 2017; Antwis and Harrison, 2018). However, quantitative estimates of the ability of multi-species consortia to elicit wide-spectrum inhibition of wildlife pathogens is scarce. Here, we test the ability of multi-isolate bacterial consortia to inhibit isolates of the chytrid fungus *Batrachochytrium dendrobatidis* (*Bd*), which causes the lethal disease chytridiomycosis in amphibians (Lips, 2016). Chytridiomycosis has resulted in the decline of over 500 amphibian species of the last 50 years (Scheele et al., 2019), and remains a major threat to amphibian biodiversity around the globe (Lips, 2016; O’Hanlon et al., 2018; Scheele et al., 2019). Application of bacterial probiotics can reduce the growth of *Bd in vitro* (Antwis et al., 2015; Muletz-Wolz et al., 2017; Piovia-Scott et al., 2017; Antwis and Harrison, 2018) and reduce *Bd*-associated mortality *in vivo* (Kueneman et al., 2016). Previous work has highlighted that constructing probiotic consortia that maximize the genetic distance among bacterial isolates elicits stronger inhibition against *Bd* than consortia containing the same number of more closely related isolates (Antwis and Harrison, 2018). These data suggest that the emergent functional properties of a probiotic consortium, in this case pathogen inhibition, may be a function of overall genetic diversity rather than species diversity (Piovia-Scott et al., 2017) and resonates with biodiversity-function relationships studies in macroecology (e.g., Brophy et al., 2017, see also Koskella et al., 2017). Maximizing intra-consortium genetic diversity may therefore increase the probability of obtaining broad-spectrum inhibition across a diverse suite of pathogen genotypes and minimize the impact of lethal wildlife diseases. Moreover, selecting bacteria that exhibit strong inhibitory capabilities individually may maximize the potential for broad-spectrum inhibition across pathogen genotypes when combined in a consortium.

Here we examine patterns of inhibition of both individual bacterial isolates and consortia tested against a panel of nine isolates of *Bd* (Table 1), comprising eight isolates of the highly virulent Global Panzootic Lineage (*Bd*GPL) and one isolate of the more distantly related *Bd*CAPE (see O’Hanlon et al., 2018). We seek to test the following predictions: (i) *Bd* isolates will vary in their susceptibility to inhibition by individual bacterial isolates, but using bacterial consortia will reduce the among-isolate variation in inhibition; (ii) probiotic consortia with higher mean genetic diversity will more likely elicit broad-spectrum inhibition of multiple pathogen variants; and (iii) *Bd* isolates that are more genetically similar will have more similar inhibition profiles across the panel of isolates tested.

**TABLE 1:** *Batrachochytrium dendrobatidis* (*Bd*) strain information for nine isolates used in this study, comprising eight isolates of BdGPL, and one of BdCAPE.

**Isolate**	**Lineage**	**Geographic location**	**Host species**	**Collector**	**Year**
CORN2.3	GPL	Penhale Farm, Cornwall, United Kingdom	*Ichthyosaurus alpestris*	Trenton Garner	2012
IA2011	GPL	Ibon Acherito, Spain	*Alytes obstetricans*	Matthew Fisher	2011
IA42	GPL	Ibon Acherito, Spain	*Alytes obstetricans*	Trenton Garner	2004
JEL423	GPL	Guabal, Panama	*Agalychnis lemur*	Joyce Longcore	2004
MG04	GPL	Silver Mine, Western Cape, South Africa	*Amietia fuscigula*	Trenton Garner	2010
MG09	GPL	Magoebaskloof, Limpopo, South Africa	*Hadromophryne natalensis*	Che Weldon	2008
MODS 28.1	GPL	Mont Olia, Sardinia, Italy	*Discoglossus sardus*	Trenton Garner	2010
SA4c	CAPE	Pinetown, KwaZulu Natal, South Africa	*Amietia angolensis*	Trenton Garner	2010
SFBC019	GPL	Sellafield, Cumbria, United Kingdom	*Epidalea calamita*	Peter Minting	2010

## Materials and Methods

### Isolation of Bacteria and Probiotic Design

We isolated bacteria from the skin of wild *Agalychnis* spp. frogs at Las Cuevas Research Station in the Maya Mountains of Belize by permission of the Belize Forestry Department (Research and Export Permit Number CD/60/3/12; Antwis et al., 2015). Briefly, we swabbed frogs and streaked these out on R2A agar. Bacteria were left to grow at ambient temperature for eight days, and we picked individual colonies using sterile swabs, which were stored in R2A media and shipped to the United Kingdom (DEFRA Authorization Number TARP/2012/224). We confirmed purity of bacteria by re-streaking on R2A and used colony PCR to amplify the 16S rRNA gene (with primers 27F and 1492R), which were sequenced at the University of Manchester. We aligned the forward and reverse sequences for each bacterium and blasted these against the NCBI database^1^ to identify bacteria. We maintained frozen stocks of pure bacteria stored in 30% glycerol and 70% tryptone solution at −80°C, which were used for subsequent *in vitro* challenge assays.

For *in vitro* challenge assays, we used three bacteria from each of four genera (*Acinetobacter* spp., *Chryseobacterium* spp., *Serratia* spp., and *Stentrophomonas* spp.) based on preliminary screening of inhibitory capabilities; medium to strong inhibitors pf *Bd*’s growth were selected to maximize the potential inhibitory capabilities of consortia (Supplementary Table S1). We added 50 μl of the 12 bacteria (three cultures per bacterial strain) to 15 ml of 1% tryptone and incubated these at 18°C for 36 h until turbid. We did not adjust cell density prior to inhibition to avoid altering the metabolite profiles already produced by cultures through the addition of media.

#### Challenge Assays

For each probiotic, we added bacteria to 12 ml of fresh 1% tryptone broth and left these to incubate for 4 h. A total of 3 ml of bacteria were used for each probiotic, split evenly between the number of bacteria added to each group; either one (“single probiotic”), two (“double probiotic”), or three (“triple probiotic”). Double probiotics were always constructed from two unique congeneric bacterial isolates. Triple probiotics were constructed from three bacteria either within the same genus (“inter-genera triple probiotic,” total of four mixes) or across three different genera (“intra-genus triple probiotic,” total of four mixes; Supplementary Table S1).

Nine *Bd* isolates were used for *in vitro* challenges, isolated from a range of host species from Europe, Africa and Central America (Table 1; see *Bd Sequencing* below for additional information). These were grown in 1% tryptone broth until maximum zoospore production was observed (∼3 days; ∼1 × 10^6^ zoospores ml^–1^). Three flasks per *Bd* isolate were grown and combined for *in vitro* challenges. *Bd* zoospores were separated from sporangia by filtering through 20 μl sterile filters (Millipore, Ireland). Bacterial probiotics were filtered (0.22 μm sterile filter; Millipore, Ireland) to isolate bacterial products in the filtrate, which were kept on ice until *Bd* challenges were conducted.

To conduct the *in vitro* challenge assays, 50 μl of bacterial filtrate and 50 μl of *Bd* suspension were combined in 96-well plates. Each *Bd* -bacteria combination was run in triplicate. Positive controls were included using 50 μl 1% tryptone instead of bacterial filtrate. Negative controls were included using 50 μl sterile tryptone and 50 μl of heat-treated *Bd* for each isolate. The optical density (OD) was measured at 492 nm filter on a spectrophotometer on initial construction of the challenge assays, and every 24 h after for four subsequent days. For each measurement, data were transformed using the equation Ln[OD/(1-OD)], and a regression analysis was used to gain the slope values for each sample over time (Antwis and Harrison, 2018). The slopes of the three replicates for each *Bd*-bacteria combination were averaged, and the slope of the negative controls subtracted. Total *Bd* inhibition was calculated using the formula: Inhibition (%) = [1−(slope of sample/slope of control)] × 100 to give an “inhibition score.” A positive inhibition score represents inhibition of *Bd* growth and a negative score indicates enhanced growth of *Bd*. We did not use a nutrient depleted control in our experiments (Bell et al., 2013), which means *Bd* inhibition relative to the controls may be slightly under-estimated.

### *Bd* Sequencing

Extracted high molecular weight DNA of six *Bd* isolates were sequenced on an Illumina HiSeq^TM^ platform (Illumina, CA, United States), which produced 125 + 125 bp paired-end sequenced reads using the Illumina HiSeq^TM^ high output V4 chemistry. Data analysis was done using a previously defined *Bd* specific alignment pipeline and stringent variant discovery conditions (O’Hanlon et al., 2018). A pairwise *Bd* relatedness matrix was generated in R by comparing genotype calls across all segregating sites in the isolate collection (185290 segregating sites).

### Statistical Analysis

All statistical analyses, code and data files are provided as a supplementary R Markdown document to allow full reproducibility of results. All statistical analyses were conducted in the software R v.3.5.3 (R Core Team, 2019). We used the Bayesian mixed effects modeling package *brms* (Bürkner, 2017, 2018) to fit statistical models. We assessed convergence of models based on R-hat values, and visually inspected chains to ensure adequate mixing. We used an information criterion based on leave-one-out cross validation (LOO-IC; Vehtari et al., 2017), calculated by the *loo* package (Vehtari et al., 2019) called via *brms* to select among candidate models, and present mean parameter estimates of effects and 95% credible intervals as appropriate. LOO-IC values presented represent comparison to the null model.

Patterns of Inhibition Across Consortium Types and *Bd* Isolates: To examine the effects of probiotic type (single isolate, double isolate, etc.), *Bd* isolate and bacterial genus on inhibition scores, we fitted a linear mixed effects model (LMM) with inhibition as the response, probiotic type as a fixed effect, and *Bd* isolate and bacterial genus as random intercepts. As the dataset contained both positive and negative inhibition values, where a negative value represents facilitation of *Bd* growth, we used a Gaussian error structure to avoid having to remove the informative negative values. Ideally, we would have fitted a 2-stage Hurdle model to such data, simultaneously modeling the factors influencing the probability of *Bd* growth facilitation, and the magnitude of *Bd* inhibition | a positive inhibition value. However, these models were regrettably too complex for this dataset as the negative values were sparse and clustered within certain *Bd* isolates. Using a Gaussian model allowed us to capture these same patterns in a simpler model. We used the R package *pheatmap* (Kolde, 2019) version 1.0.12 to visualize differences in *Bd* inhibition across all probiotics tested.

#### *Bd* Relatedness and Inhibition

To assess whether more closely related *Bd* isolates had more similar inhibition profiles, we conducted a Mantel test on a matrix of pairwise relatedness values among the six *Bd* isolates for which we had genomic data, and a pairwise matrix where each cell value represented the correlation between the inhibition profiles for each pair of *Bd* isolates. To build a tree of *Bd* isolates, we used hierarchical clustering of genetic distance values using the “upgma” function in the *phangorn* package (Schliep, 2011; Schliep et al., 2017) in R.

#### Multi-Isolate Probiotics and Broad-Spectrum *Bd* Inhibition

To calculate genetic distance among bacterial isolates, we aligned the 16S rRNA gene sequences against the SILVA v132 references alignment (Quast et al., 2013; Yilmaz et al., 2014) using *mothur* 1.39.5 (Schloss et al., 2009). We used the *seqinr* package (Charif and Lobry, 2007) to read the alignment into R and calculate inter-sequence distance. To examine the effect of mean genetic distance among consortium members on *Bd* inhibition, we fitted a LMM with *Bd* inhibition as a response, both *Bd* isolate and probiotic genus as random intercepts, and genetic distance as a random slope given *Bd* isolate (i.e., *Bd*-specific slopes for the effect of probiotic genetic distance). As so many inhibition values were near the upper bound (100% inhibition), we logit transformed inhibition scores after converting % scores to the scale (0,1), and used a Gaussian error structure (Warton and Hui, 2011).

## Results

### Patterns of Inhibition Across Consortium Types and *Bd* Isolates

**FIGURE 1:**
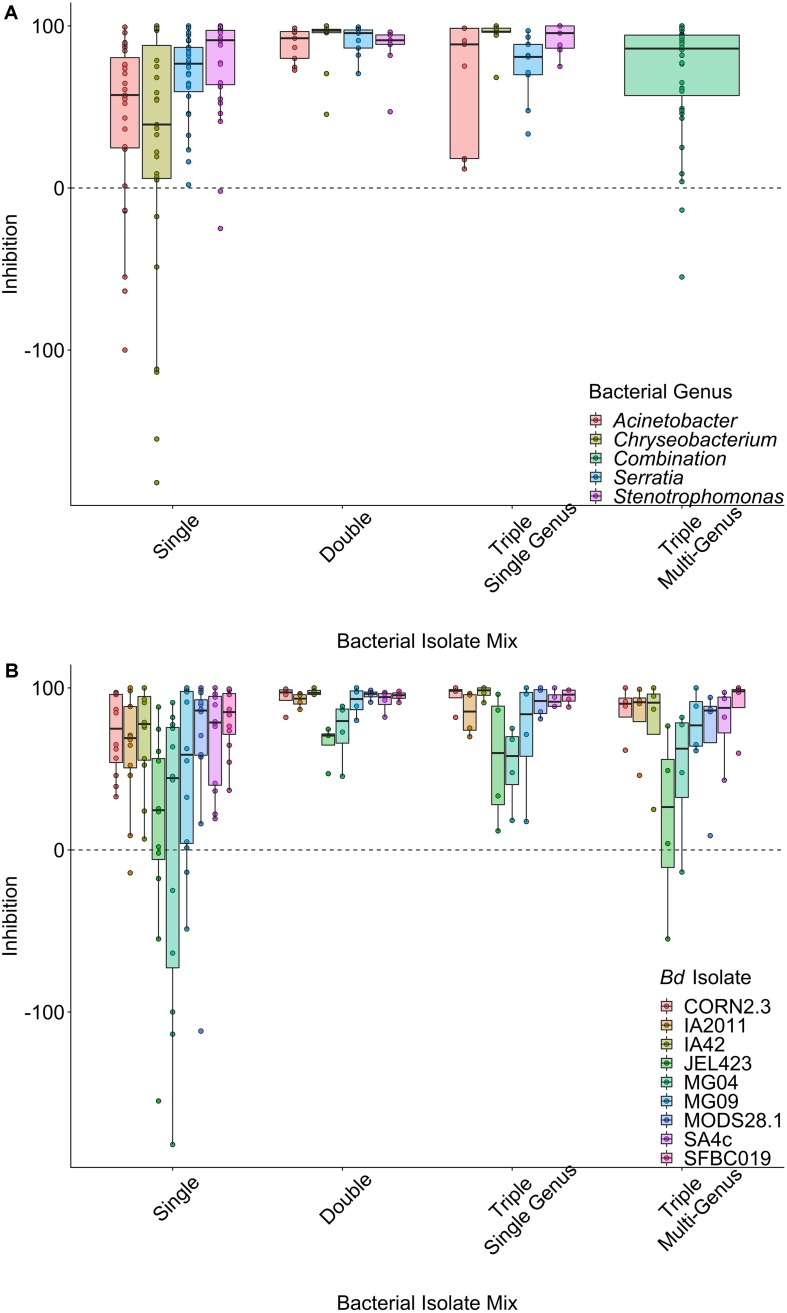
Raw data plots of inhibition scores split by probiotic type, grouped by **(A)** bacterial genus averaged over *B. dendrobatidis* (*Bd*) isolates and **(B)** *Bd* isolates averaged over bacterial genus. Data are displayed as boxplots with raw data points overlaid. Positive values (above the dashed line) indicate inhibition of *Bd* growth, whereas negative values indicate that *Bd* grows faster in the presence of the probiotic. There is clear variation in inhibition strength across *Bd* isolates and probiotic types.

There was strong variation in patterns of inhibition among both probiotic types (Figures 1A,B) and among *Bd* isolates within probiotic types (Figure 1B). When controlling for the effects of *Bd* isolate identity and bacterial genus in a LMM, single-isolate probiotics exhibited the lowest average inhibition scores (effect of probiotic type; ΔLOO = −14.3; Figures 1A,B). The model explained 33.1% (95% credible interval, 24.1% to 41.1%) of the variance in inhibition scores overall. Considering only the random effects, variance component analysis indicated that *Bd* isolate explained slightly more variance than bacterial genus [*Bd* mean (95% CI) = 28.4% (16.1–40.7); bacterial genus mean = 21.1% (7.9–36.3)].

**FIGURE 2:**
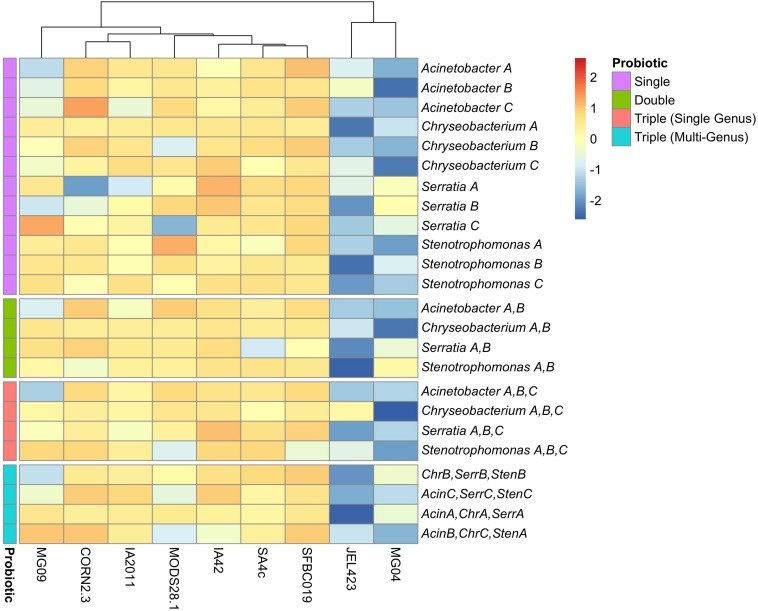
Heatmap of inhibition scores across nine *Bd* isolates for all probiotic types. Rows are ordered based on increasing probiotic complexity, from single bacterial isolate to multi-genus triple-isolate probiotics. Probiotics are color-coded according to complexity (see “Probiotic” legend). Columns have been clustered according to similarity of inhibition profiles among *Bd* isolates. Data are scaled by row to permit comparison of inhibition strength across *Bd* isolates for each probiotic. Multi-isolate probiotic abbreviations: *Chr*, Chryseobacterium; *Acin*, Acinetobacter; *Serr*, Serratia; *Sten*, Stenotrophomonas.

**FIGURE 3:**
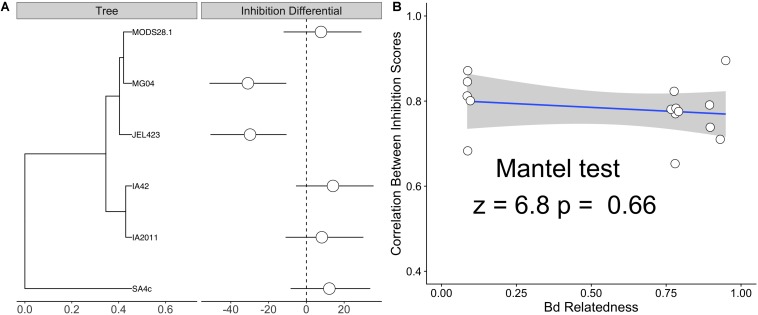
**(A)** Phylogenetic tree of six sequenced *Bd* isolates alongside isolate-specific inhibition differential – defined as the departure from the global mean inhibition value from a Bayesian linear mixed effects model (LMM) with a *Bd* random effect. Estimates are marginalized with respect to bacterial genus of probiotic isolates. Both JEL423 and MG04 exhibit 95% credible intervals not crossing zero, indicative of lower average inhibition potential compared to other isolates. **(B)** Correlation plot between the average pairwise relatedness between *Bd* isolates and the correlation between their inhibition scores for the panel of tested probiotics (listed in Figure 2). A Mantel test indicated no obvious relationship between inter-isolate genetic distance and similarity of inhibition profiles (*p* = 0.6).

We also detected substantially lower average inhibition for two of the nine *Bd* isolates (JEL423 and MG04) irrespective of the probiotic type applied (Figure 2 and Supplementary Figure S1). Hierarchical clustering also identified these two *Bd* isolates as having divergent inhibition profiles compared to other seven isolates (Figure 2). We found no evidence that more closely related *Bd* isolates had more similar inhibition profiles across the panel of probiotics tested (Mantel test *p* = 0.6; Figures 3A,B), although the two *Bd* isolates most resistant to inhibition were relatively genetically similar (both isolates were BdGPL JEL423/MG04 with a pairwise relatedness = 0.89; Figure 3A).

### Multi-Isolate Probiotics and Broad-Spectrum *Bd* Inhibition

**FIGURE 4:**
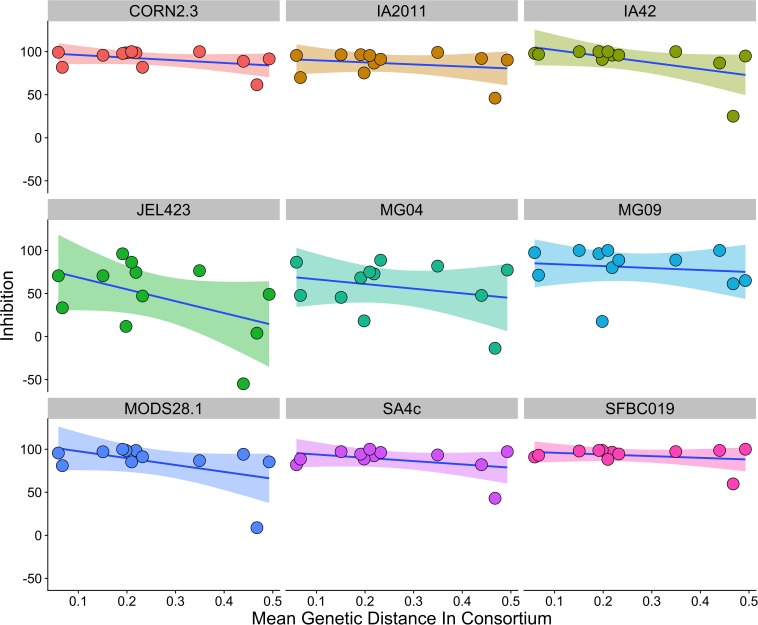
Relationship between average genetic distance of members of a multi-isolate probiotic (2 isolates or more) and inhibition of *Bd*, split by *Bd* isolate identity. There was no evidence of a relationship between genetic distance and inhibition when controlling for probiotic ID, bacterial genus and *Bd* isolate identity. A linear trend line is provided for illustration.

We found that consortia containing isolates from three different bacterial genera had higher average between-isolate distance than those containing two or three bacterial isolates from the same genus (Supplementary Figure S2). However, there was no evidence that higher average genetic distance among isolates in a probiotic resulted in stronger inhibition when controlling for *Bd* isolate and bacterial genus [effect of genetic distance mean (95% CI) = −2.77 (−10.99,5.09); ΔLOO effect of genetic distance = −0.2; Figure 4].

**FIGURE 5:**
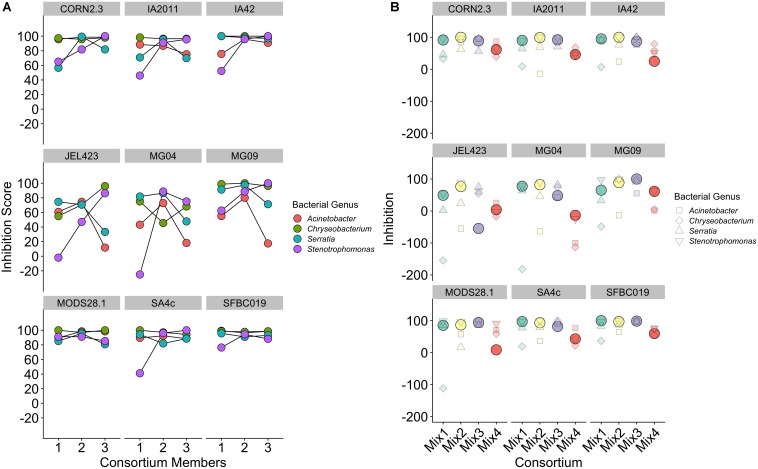
**(A)** Inhibition scores for single-genus probiotics of increasing complexity from single to three-isolate consortia, split by *Bd* isolate. **(B)** Inhibition scores for three-member probiotic consortia containing isolates from three different bacteria genera. Solid circles indicate the inhibition score for a consortium for each *Bd*. Smaller shapes show the individual inhibition scores of the three single isolates that comprise each consortium, with shape corresponding to bacterial isolate genus.

Considering only consortia constructed using bacterial isolates from a single genus, we detected no consistent effect of increasing probiotic complexity from one to three isolates on overall inhibition score [effect of number of isolates, mean (95% CI) = 3.66 (−0.86; 8.06); Figure 5A]. Conversely, variance component analysis revealed strong variation among both bacterial genera and *Bd* isolates in determining overall inhibition [percentage variance explained (95% CI): *Bd* = 34.96 (19.3–51.3); bacterial genus = 21.68 (2.73–44.6)]. The high variance among *Bd* isolates is evidenced by three *Bd* isolates (JEL423, MG04, and MG09) displaying highly unstable inhibition dynamics as probiotic complexity increased (Figure 5A). Whereas for most *Bd* isolates, increasing probiotic complexity yielded higher or stable inhibition scores, for JEL423, MG04, MG09 the addition of bacterial isolates appeared to cause decreases in average inhibition for two (JEL423 and MG09) and three (MG04) of the bacterial consortia, respectively. Consistent with this, there was a negative correlation across all *Bd* isolates between the mean inhibition and variance in inhibition across genera for three-isolate probiotics (Spearman’s rho = −0.93, *p* < 0.001; Figure 5A).

Considering only probiotics constructed using bacterial isolates from multiple genera, in 72% of cases (26/36) the probiotic exhibited an inhibition score that was higher than the mean of the single-isolate inhibition scores for the isolates that probiotic contained (Figure 5B; Wilcoxon signed rank test, *V* = 514, *p* = 0.004). Only in 19% of cases (7/36) did the inhibition score exceed the score of the isolate with strongest inhibition in the probiotic (Wilcoxon signed rank rest *V* = 66.5, *p* = 1; Figure 5B). As for single-genus triple-isolate probiotics, we observed a negative correlation between the among-consortium variance in inhibition and mean inhibition across the nine *Bd* isolates (Spearman’s rho = −0.86, *p* < 0.005; Figure 5B).

## Discussion

Our findings have revealed consistent variation among *Bd* isolates in their susceptibility to inhibition by potential bacterial probiotics. Whilst the growth of most isolates could be suppressed by between 70 and 100%, two of the isolates (JEL423 and MG04) appeared to resist inhibition irrespective of the probiotic type being employed. Consistent with previous research, treatments comprising just a single bacterial isolate performed worse on average when tested across a range of *Bd* isolates (Antwis et al., 2015; Muletz-Wolz et al., 2017; Antwis and Harrison, 2018). Employing multi-isolate treatments increased average inhibition, but contrary to expectation, increasing the genetic diversity of the consortium did not lead to increased inhibition values. These data have important ramifications for our ability to design probiotics that are consistently effective against the diverse genetic variants of *Bd* encountered in nature (Becker et al., 2017; O’Hanlon et al., 2018).

There is now growing evidence that multi-strain probiotic consortia offer both increased inhibition potential for individual *Bd* isolates (Piovia-Scott et al., 2017), and a higher probability of effecting broad-spectrum inhibition across multiple genomic variants of *Bd*, compared to probiotics comprising only a single bacterial isolate (Antwis and Harrison, 2018; this study). Using probiotics containing two or more isolates impeded *Bd* growth more strongly on average than single isolates. However, unlike a previous study (Antwis and Harrison, 2018), we found no support for our prediction that more genetically diverse probiotic consortia were more effective at constraining *Bd* growth. One explanation for this pattern is that the effectiveness of two-isolate consortia was already very high because consortia were constructed from isolates known to offer strong inhibition against one of more *Bd* isolates. It follows, therefore, that when inhibition by single bacterial isolates is weak, co-culturing genetically diverse bacteria can elicit much stronger inhibition (Antwis and Harrison, 2018). However, when variance in inhibition is compressed because average inhibition is already high, the genetic distance among consortium members is less important in determining inhibition strength. The corollary of this is that there will undoubtedly be variants of *Bd* even more resistant to treatment with probiotics not tested in this dataset, and in such cases where mean growth inhibition is lower (i.e., yielding more variance in the trait) we might expect the diversity-function relationship to re-emerge.

Though we observed an increase in mean inhibition when using two more bacterial isolates in a consortium, the dynamics of change in inhibition with increasing probiotic complexity were not uniform across isolates of *Bd*. For three isolates in particular (JEL423, MG04, and MG09), increasing the species-richness of the probiotic destabilized inhibition dynamics and increased variance in inhibition among bacterial genera. It is noteworthy that two of these isolates were those that were most resistant to inhibition; we observed the same negative mean-variance relationship for consortia comprising isolates from three bacterial genera as we did for single-genus consortia. Similarly, it was rare to observe inhibition values for triple consortia that exceeded that of the strongest single isolate inhibitor in the community, indicating no evidence for strong synergistic effects when co-culturing. Taken together, these results suggest that the emergent functional properties of these microbial communities, specifically the efficacy of the repertoire of anti-fungal metabolites they produce when co-cultured, vary in concert with the genotype of the pathogen. These patterns highlight the difficulties associated with predicting microbial community dynamics and function purely from species composition (Widder et al., 2016; Momeni et al., 2017; see also Koskella et al., 2017). Previous work using consortia to mitigate *Bd* has observed strong dominance effects in probiotic communities, where the species attaining highest relative abundance following co-culture is determined by the initial species composition (Piovia-Scott et al., 2017). Though we did not track relative abundance of individual community members, variation across the consortia we tested relative to their individual isolate inhibition scores likely reflects competition effects driving different members of each consortium to dominance (see Friedman et al., 2017). Clearly, increasing probiotic complexity can modify emergent function in either a beneficial or a detrimental way, depending on the *Bd* isolate used in the challenge. Therefore, the outcome of the competition changes with total community complexity (e.g., Piovia-Scott et al., 2017), which in turn modifies the utility of the consortium to depress *Bd* growth. Elucidating these complex relationships between microbial community diversity-function and pathogen genotype forms a critical component of our understanding of how to develop probiotics with maximum effectiveness in the face of enormous pathogen genomic variation. More broadly, it highlights the utility of the bacteria-*Bd* system for answering general questions about how community function scales with complexity in microbial assemblages.

We found no evidence that genetic similarity of *Bd* isolates predicted similarity of inhibition scores across the six isolates for which there were genomic data, comprising five *Bd*GPL isolates and one *Bd*CAPE. Muletz-Wolz et al. (2017) found that the inhibition profiles of eight *Bd* isolates clustered by phylogenetic relatedness, but tested a much larger phylogenetic breath of isolates including *B. salimandrivorans* (*Bsal*) and *B. dendrobatidis-Brazil*, which clustered together and separately from six *Bd*GPL isolates. We detected no such distinction in our panel of *Bd*s, where we might have expected the *Bd*CAPE lineage to cluster independently of isolates belonging to *Bd*GPL. Whilst our data do not support a linear trend between relatedness and inhibition, we cannot discount the possibility that there may be clusters of resistant *Bd* genotypes throughout the broader *Bd* phylogeny. *Bd* can rapidly evolve resistance to inhibition by antimicrobial peptides through changes in genomic traits such as chromosomal copy number (Farrer et al., 2013), and the presence of two inhibition-resistant isolates in our dataset may reflect prior selection against constraint of their growth by bacterial metabolites. Future work should seek to quantify susceptibility to inhibition over a much broader repertoire of *Bd* lineages, including *Bsal*, to test this possibility. It would be particularly interesting to investigate whether clusters of inhibition-resistant genotypes cluster spatially and/or align with particular environmental conditions. Recent *in vitro* work has highlighted that abiotic conditions such as temperature can both affect the efficacy of bacterial probiotics against *Bd* (Muletz-Wolz et al., 2017) and virulence traits of the pathogen such as growth rate of zoosporangia (Muletz-Wolz et al., 2019). These studies suggest that although we have achieved relatively consistent inhibition across multiple pathogen genomic variants in this study under controlled laboratory conditions, the true emergent properties of probiotics in nature will instead be function of pathogen genome, probiotic genome(s), and environment interactions (GxGxE), which makes predicting inhibition strength of these probiotics for novel *Bd* genotypes even more challenging.

In our study, we filtered metabolites derived from bacterial co-culture to be used in inhibition challenges against *Bd*. Though challenges of this nature provide compelling evidence of the ability of certain bacterially derived metabolites to prevent *Bd* growth, they are not necessarily accurate reflections of natural interactions among host-associated microbes, or between microbes and the pathogen. For example, the phenotypic traits of bacteria grown in planktonic form, as we have cultured them, can differ vastly from those same bacteria grown in biofilms (e.g., San-Wong et al., 2015). Likewise, our assays prevent us from quantifying how the strength of bacteria-Bd interactions may be modified by presence of physical contact between cells of the resident bacteria and the pathogen. One way to overcome these limitations for future work would be to use a biolfilm model of infection, which is ideally suited to studying both polymicrobial dynamics and pathogen interactions (Gabrilska and Rumbaugh, 2015). Such assays would provide more accurate estimates of competition and growth processes within bacterial communities, and permit physical interaction between bacterial biofilms and *Bd*. Concordant with our results, Piovia-Scott et al. (2017) used a biofilm model to demonstrate that the strength of inhibition of *Bd* is a function of increased bacterial community complexity (see also Antwis and Harrison, 2018). Future work should seek to quantify the metabolic phenotype of bacteria grown in biofilm conditions to identify the functional mechanism by which host-associated microbes can mitigate the proliferation of the pathogen.

In summary, developing effective probiotic strategies to mitigate wildlife disease is clearly complicated by the ability of the pathogen to evolve resistance to potential probiotics (Farrer et al., 2013), the modulation of pathogen virulence and probiotic effectiveness by abiotic conditions (Muletz-Wolz et al., 2017, 2019), and the ability of the pathogen to generate novel, potentially more virulent genotypes through hybridization (Greenspan et al., 2018). Understanding the interplay between pathogen genomics and local environment in determining pathogen virulence, and the ability to dampen virulence traits with probiotics, remains a major research priority.

## Data Availability Statement

All code and data to reproduce the results in this manuscript are available on Figshare at DOI https://doi.org/10.6084/m9.figshare.c.4666922.

## Ethics Statement

The animal study was reviewed and approved by the University of Salford Ethical Approval Committee.

## Author Contributions

RA and XH conceived the study and wrote the manuscript with input from all authors. RA collected the bacterial inhibition data. TS and MF sequenced the *Bd* isolates and calculated *Bd* relatedness data. XH analyzed the data.

## Conflict of Interest

The authors declare that the research was conducted in the absence of any commercial or financial relationships that could be construed as a potential conflict of interest.

## Footnotes

1 http://blast.ncbi.nlm.nih.gov/Blast.cgi
