# Supplementary material for: Designing Probiotic Therapies With Broad-Spectrum Activity Against a Wildlife Pathogen
Source: Front Microbiol. 2020 Jan 22;10:3134. doi: 10.3389/fmicb.2019.03134 (PMC6987264; doi:10.3389/fmicb.2019.03134)
Supplement: Supplementary file 2 [file Data_Sheet_2.docx]

# SUPPLEMENTARY INFORMATION: Designing Probiotic Therapies with Broad-Spectrum Activity Against a Wildlife Pathogen

Xavier A. Harrison^1,2#^, Thomas Sewell^3^, Matthew Fisher^3^, Rachael E. Antwis^4 #^

### FIGURE S1

Conditional modes of random effects from a linear mixed model examining effect of probiotic type on inhibition of *Bd.* (A) Inhibition differential (departure from global mean inhibition value) for each of 9 *Bd* isolates. (B) Inhibition differentials for 4 bacterial genera and the combination category representing multi-genus probiotic consortia.

### FIGURE S2

Violin plot of genetic distance among probiotic consortium members. Probiotics containing three isolates from different genera had higher mean inter-isolate genetic distance compared to probiotics containing two (Double) or three (Triple) isolates from the same genus
